# Right Frontal Isocitrate Dehydrogenase (IDH)-Mutant WHO Grade 3 Oligodendroglioma Presenting With Morning Headache and Gait Imbalance

**DOI:** 10.7759/cureus.110633

**Published:** 2026-06-10

**Authors:** Laila Metwaly

**Affiliations:** 1 Neurology, Private Practice, New York City, USA

**Keywords:** 1p/19q codeletion, frontal lobe tumor, idh-mutant glioma, morning headache, neuro-oncology, oligodendroglioma, who grade 3 glioma

## Abstract

Oligodendroglioma is a diffuse glioma characterized by isocitrate dehydrogenase (IDH) mutation and 1p/19q codeletion. We report the case of a 45-year-old woman who presented with progressive headaches, nausea, vomiting, blurred vision, and gait imbalance associated with rapid neurological deterioration. Neuroimaging demonstrated a large right frontal intra-axial lesion with heterogeneous enhancement, cystic components, surrounding edema, and significant mass effect with midline shift. The patient underwent subtotal surgical resection, and histopathological evaluation confirmed an IDH-mutant, 1p/19q-codeleted oligodendroglioma. Postoperatively, she received adjuvant chemoradiotherapy followed by temozolomide, with subsequent clinical and radiographic improvement. This case highlights the importance of early recognition of progressive neurological symptoms and multidisciplinary management in patients presenting with aggressive intracranial tumors.

## Introduction

Oligodendrogliomas are rare primary central nervous system neoplasms originating from oligodendroglial lineage cells and represent a minority of adult diffuse gliomas [1,2]. Historically, these tumors were classified primarily according to histopathological characteristics; however, advances in molecular neuro-oncology have substantially revised their diagnostic criteria. The 2021 World Health Organization (WHO) Classification of Tumors of the Central Nervous System now requires the presence of an isocitrate dehydrogenase (IDH) mutation together with whole-arm 1p/19q codeletion to establish the diagnosis of oligodendroglioma as a distinct molecular entity [1,3]. This integrated molecular approach has replaced earlier morphology-based systems and has improved prognostic stratification, therapeutic planning, and diagnostic precision in diffuse gliomas [1,4,5]. IDH-mutant and 1p/19q-codeleted oligodendrogliomas are generally associated with improved treatment responsiveness and overall survival compared with IDH-wildtype diffuse gliomas according to the WHO 2021 molecular classification criteria [1]. However, WHO grade 3 oligodendrogliomas demonstrate increased mitotic activity, greater growth potential, and more aggressive biological behavior than lower-grade oligodendroglial tumors, often resulting in increased neurological morbidity and risk of progression. 

In adults, oligodendrogliomas most commonly arise in the cerebral hemispheres and demonstrate a predilection for the frontal lobes [2,6]. Clinical manifestations vary depending on tumor location, growth kinetics, and mass effect and may include seizures, cognitive or behavioral changes, headaches, and focal neurological deficits such as gait imbalance [2]. Higher-grade oligodendrogliomas frequently exhibit more aggressive biological behavior, including rapid symptom progression, peritumoral edema, contrast enhancement, and significant midline shift on neuroimaging [6,7]. These clinical and radiographic findings emphasize the importance of prompt diagnostic evaluation, particularly in patients presenting with progressive morning headaches and evolving neurological dysfunction.

Current management strategies emphasize maximal safe surgical resection followed by adjuvant radiotherapy and chemotherapy, commonly involving temozolomide or procarbazine, lomustine, and vincristine (PCV)-based regimens [2,8]. Although IDH-mutant and 1p/19q-codeleted oligodendrogliomas generally confer a more favorable prognosis than IDH-wildtype diffuse gliomas, grade 3 tumors may still demonstrate clinically aggressive behavior requiring long-term multidisciplinary management [7,9-11].

This report describes a middle-aged woman presenting with worsening morning headaches and gait imbalance secondary to a right frontal IDH-mutant, 1p/19q-codeleted WHO grade 3 oligodendroglioma. The case highlights the diagnostic relevance of symptom recognition, advanced neuroimaging, and molecular characterization in guiding optimal management decisions. This case was previously presented as a poster at the Texas Neurology Society Winter Meeting 2026 held in Texas, USA, in January 2026.

## Case presentation

A 45-year-old right-handed woman with a past medical history of hypothyroidism presented to the emergency department with a one-week history of progressively worsening morning headaches. The headaches were described as dull, pressure-like, and predominantly frontal, accompanied by nausea, projectile vomiting, blurred vision, and gait instability. Family members additionally reported progressive unsteadiness and difficulty initiating steps. She denied seizures, speech disturbances, or loss of consciousness.

On neurological examination, the patient was alert and fully oriented. Motor strength was preserved, and no focal sensory deficits were identified. Cranial nerve examination was grossly intact; however, fundoscopic examination revealed papilledema, consistent with elevated intracranial pressure. The patient demonstrated gait imbalance during ambulation. Given the concerning clinical findings and suspicion of elevated intracranial pressure, urgent neuroimaging was obtained, revealing a large intracranial mass with significant mass effect and midline shift, prompting expedited neurosurgical evaluation and intervention.

A noncontrast head CT revealed a large right frontal intra-axial lesion exerting significant mass effect (Figure 1). Subsequent brain MRI with contrast demonstrated a predominantly cystic right frontal neoplasm measuring 56.2 × 42.7 × 59.8 mm, characterized by irregular thick walls, heterogeneous T1/T2 signal, blooming foci suggestive of hemorrhagic components, mild perilesional edema, and a 15 mm leftward midline shift (Figure 2). Diffusion-weighted imaging revealed restricted diffusion along tumor margins, and MR spectroscopy demonstrated elevated choline peaks, consistent with high-grade glioma behavior. The patient's MRI is shown in Figure 1. Access to histopathological slide images was restricted at the time of manuscript preparation; therefore, only radiological imaging findings are presented in this report.

**Figure 1:**
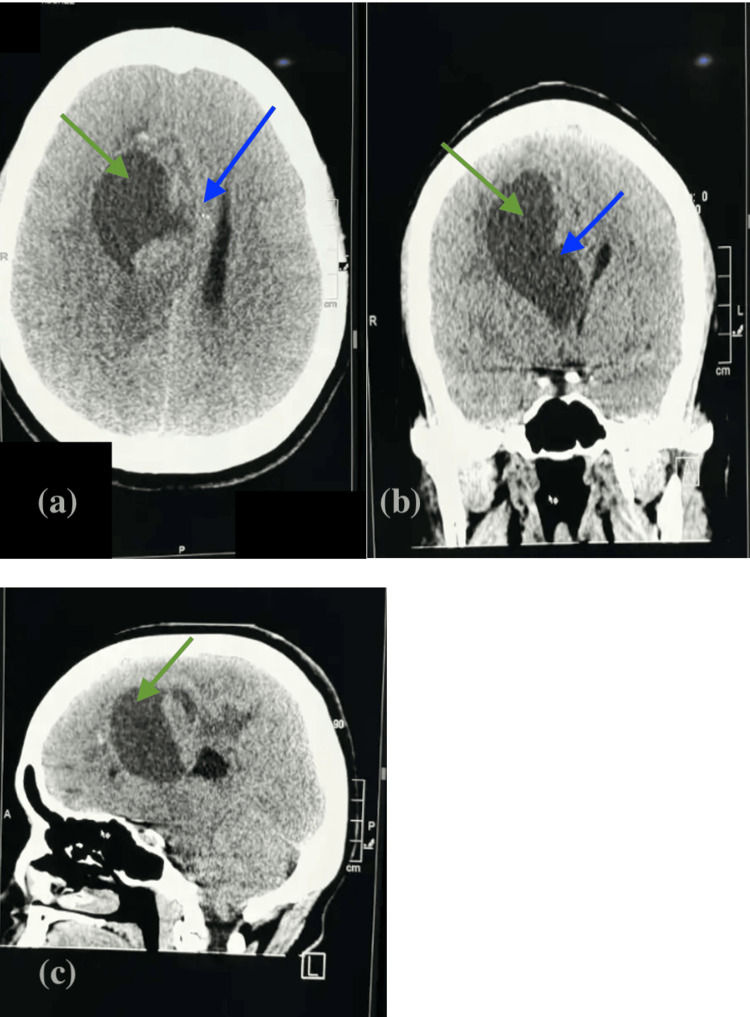
CT imaging of a right frontal cystic oligodendroglioma demonstrating mass effect and midline shift CT: computed tomography Noncontrast CT of the brain demonstrates a large right frontal predominantly cystic intra-axial lesion associated with significant mass effect, compression of the right lateral ventricle, and leftward midline shift. Green arrows indicate the primary lesion, while blue arrows indicate the associated midline shift. (a) Axial CT view demonstrating the cystic right frontal mass with ventricular compression and leftward midline deviation. (b) Coronal CT view demonstrating the lesion and associated mass effect with displacement of midline structures. (c) Sagittal CT view demonstrating the anterior-posterior extent of the lesion and its relationship to adjacent intracranial structures

**Figure 2:**
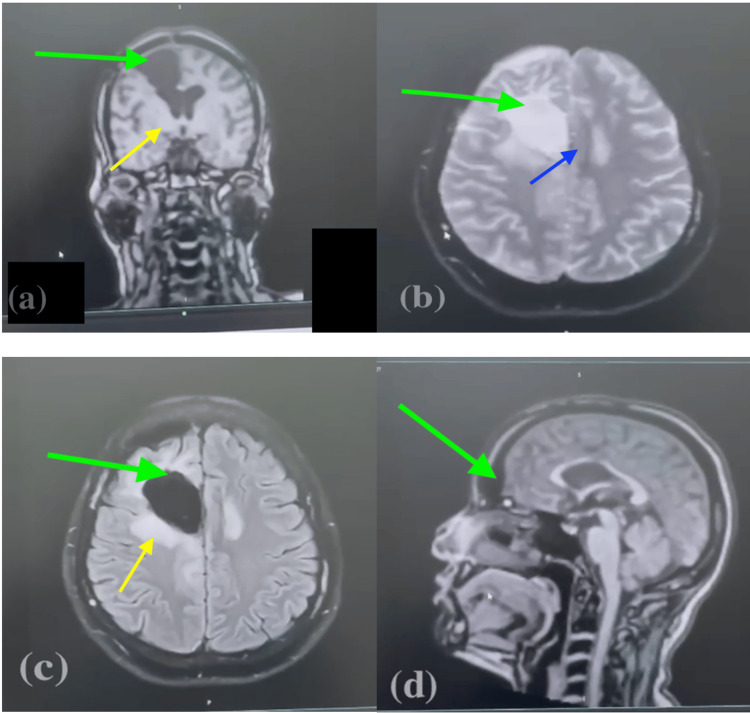
Multiplanar MRI findings of a right frontal IDH-mutant, 1p/19q-codeleted WHO grade 3 oligodendroglioma IDH: isocitrate dehydrogenase; WHO: World Health Organization; FLAIR: fluid-attenuated inversion recovery; MRI: magnetic resonance imaging Multiplanar MRI of a right frontal IDH-mutant, 1p/19q-codeleted WHO grade 3 oligodendroglioma demonstrating heterogeneous tumor signal, cystic change, diffusion restriction, vasogenic edema, and mass effect. Green arrows indicate the primary tumor lesion, yellow arrows indicate surrounding vasogenic edema, and the blue arrow indicates leftward midline shift. (a) Coronal T1-weighted MRI demonstrating a right frontal parasagittal lesion involving the superior frontal lobe adjacent to the falx cerebri, with associated mass effect and surrounding edema. (b) Axial diffusion-weighted imaging (DWI) demonstrating a right frontal cortical-subcortical mass with diffusion restriction and associated leftward midline shift. (c) Axial FLAIR MRI demonstrating a centrally cystic component within the lesion surrounded by vasogenic edema involving the adjacent frontal white matter. (d) Sagittal T1-weighted MRI demonstrating the superior frontal parasagittal extent of the lesion with associated cortical distortion and mass effect

The patient underwent a right frontal craniotomy with subtotal tumor resection. Although subtotal resection was reported intraoperatively, postoperative MRI demonstrated no significant residual enhancing tumor. This finding likely reflects the absence of measurable enhancing residual disease rather than the complete radiological absence of nonenhancing tumor tissue.

Postoperatively, the patient recovered without new neurological deficits. Follow-up MRI with contrast and perfusion imaging demonstrated expected postoperative changes, including regression of the midline shift from 15 mm to 10 mm, reduction in perilesional edema, and no significant residual enhancing lesion. A postoperative surgical cavity was identified, and MR perfusion imaging did not demonstrate increased cerebral blood volume suggestive of residual active tumor. The patient subsequently underwent combined chemoradiotherapy followed by adjuvant temozolomide. Serial follow-up imaging demonstrated radiographic stability with continued improvement in mass effect and no evidence of significant disease progression.

At the time of manuscript preparation, more than five years after surgical treatment, the patient remained functionally independent and had returned to her usual daily and professional activities without documented neurological deterioration. No additional disease-related interventions were known to the authors, and the patient continued to maintain a good quality of life according to the available follow-up information.

## Discussion

Oligodendrogliomas constitute a distinct molecular subgroup of adult-type diffuse gliomas defined by the coexistence of IDH mutation and whole-arm 1p/19q codeletion [1,3,4]. The introduction of molecularly integrated classification systems has significantly reshaped the diagnostic and therapeutic landscape of diffuse gliomas by improving diagnostic accuracy and prognostic stratification beyond histopathological features alone [1,5]. The present case illustrates a clinically aggressive presentation of a WHO grade 3 oligodendroglioma arising within the frontal lobe, a region commonly affected because of its high concentration of susceptible glial precursor cells [2,6].

Morning-predominant headache, as observed in this patient, is a well-recognized manifestation of elevated intracranial pressure and may occur secondary to impaired cerebrospinal fluid dynamics and nocturnal increases in intracranial pressure [11]. The associated gait imbalance likely reflected frontal lobe dysfunction and increasing mass effect. Such subtle but progressive neurological symptoms should prompt early neuroimaging evaluation, especially when accompanied by nausea, vomiting, papilledema, or cognitive changes.

Radiographic characteristics in this case, including mixed cystic-solid morphology, heterogeneous enhancement, peritumoral edema, and significant midline shift, are frequently associated with higher-grade oligodendrogliomas [6,7]. Advanced imaging findings such as elevated choline peaks on MR spectroscopy further support increased membrane turnover and tumor proliferation, correlating with more aggressive tumor biology. Although IDH-mutant and 1p/19q-codeleted tumors generally demonstrate more favorable outcomes than IDH-wildtype astrocytomas or glioblastomas, grade 3 oligodendrogliomas may nonetheless exhibit rapid clinical progression and substantial mass effect, as demonstrated in this patient [7,8]. A limitation of this report is the inability to include histopathological slide images because access to the pathology material was restricted during manuscript preparation.

Maximal safe surgical resection remains the cornerstone of treatment for oligodendrogliomas and serves both diagnostic and therapeutic purposes [2,9]. In the present case, subtotal resection was considered appropriate given the lesion size, location, and surrounding anatomical constraints. Adjuvant chemoradiotherapy plays an essential role in improving progression-free and overall survival in patients with high-grade oligodendrogliomas, particularly in tumors harboring IDH mutation and 1p/19q codeletion [2,10]. Combined treatment approaches using radiotherapy with temozolomide or PCV-based chemotherapy have demonstrated favorable long-term outcomes and remain consistent with current neuro-oncology management guidelines [2,9,10].

In this case, the available oncology records documented treatment with external-beam radiotherapy and temozolomide, followed by adjuvant temozolomide. The rationale for selecting a temozolomide-based regimen over a PCV-based regimen was not explicitly documented in the accessible records. Nevertheless, temozolomide-based chemoradiotherapy is commonly utilized in clinical practice because of its oral administration, simplified dosing schedule, and generally more favorable toxicity and tolerability profile. In contrast, although PCV remains an important evidence-supported regimen for patients with IDH-mutant, 1p/19q-codeleted WHO grade 3 oligodendroglioma and is favored by some contemporary guidelines, its use may be limited by a greater treatment burden and higher rates of hematologic and constitutional adverse effects. Therefore, treatment selection is often individualized according to patient characteristics, tolerability considerations, institutional practice patterns, and multidisciplinary decision-making.

Consistent with the established behavior of IDH-mutant, 1p/19q-codeleted oligodendrogliomas, our patient demonstrated favorable postoperative radiographic evolution and response to adjuvant therapy, similar to outcomes reported for molecularly favorable oligodendrogliomas. Nevertheless, this case illustrates that even molecularly favorable oligodendrogliomas may present with substantial mass effect, significant midline shift, and progressive symptoms of raised intracranial pressure requiring urgent neurosurgical evaluation. The case further highlights the value of multimodal neuroimaging, including MRI, diffusion-weighted imaging, perfusion imaging, and spectroscopy, in preoperative assessment and treatment planning.

Postoperative imaging in this patient demonstrated a reduction of midline shift and no significant residual enhancing tumor, reflecting successful early management. Nevertheless, long-term surveillance remains critical, as oligodendrogliomas retain the potential for recurrence or malignant progression despite favorable molecular features [7,8]. Molecular characterization not only guides diagnosis and prognosis but also increasingly influences follow-up strategies and eligibility for evolving targeted therapies.

This case reinforces several important clinical lessons. Progressive morning headaches and subtle gait disturbances should not be underestimated, particularly when associated with signs of increased intracranial pressure. Furthermore, accurate molecular classification is indispensable for modern glioma diagnosis and treatment planning. Early recognition, timely neuroimaging, integrated molecular diagnostics, and multidisciplinary management remain essential for optimizing outcomes in patients with high-grade oligodendrogliomas.

## Conclusions

This case highlights the importance of early recognition and evaluation of progressive neurological symptoms, particularly morning headaches and gait imbalance, which may indicate significant intracranial pathology. The diagnosis of oligodendroglioma now relies on integrated molecular criteria, including IDH mutation and 1p/19q codeletion, which remain central to accurate classification, prognostication, and treatment planning. Although many oligodendrogliomas demonstrate relatively favorable outcomes, WHO grade 3 tumors may present with aggressive radiographic features and substantial mass effect requiring prompt multidisciplinary management. Early neuroimaging, maximal safe surgical resection, and appropriate adjuvant therapy remain essential for optimizing outcomes in patients with high-grade oligodendrogliomas. Although the favorable clinical and radiographic course observed in this patient is encouraging, the findings of this report are based on a single case and should be interpreted with appropriate caution. Further studies with larger patient cohorts are needed to better characterize long-term outcomes in patients with high-grade oligodendroglioma.
